# Color and texture associations in voice-induced synesthesia

**DOI:** 10.3389/fpsyg.2013.00568

**Published:** 2013-09-02

**Authors:** Anja Moos, David Simmons, Julia Simner, Rachel Smith

**Affiliations:** ^1^School of Critical Studies, Laboratory of Phonetics, College of Arts, University of GlasgowGlasgow, UK; ^2^College of Science and Engineering, School of Psychology, University of GlasgowGlasgow, UK; ^3^Department of Psychology, School of Philosophy, Psychology and Language Sciences, University of EdinburghEdinburgh, UK

**Keywords:** voice-induced synesthesia, color, texture, cross-modal correspondence, speech acoustics

## Abstract

Voice-induced synesthesia, a form of synesthesia in which synesthetic perceptions are induced by the sounds of people's voices, appears to be relatively rare and has not been systematically studied. In this study we investigated the synesthetic color and visual texture perceptions experienced in response to different types of “voice quality” (e.g., nasal, whisper, falsetto). Experiences of three different groups—self-reported voice synesthetes, phoneticians, and controls—were compared using both qualitative and quantitative analysis in a study conducted online. Whilst, in the qualitative analysis, synesthetes used more color and texture terms to describe voices than either phoneticians or controls, only weak differences, and many similarities, between groups were found in the quantitative analysis. Notable consistent results between groups were the matching of higher speech fundamental frequencies with lighter and redder colors, the matching of “whispery” voices with smoke-like textures, and the matching of “harsh” and “creaky” voices with textures resembling dry cracked soil. These data are discussed in the light of current thinking about definitions and categorizations of synesthesia, especially in cases where individuals apparently have a range of different synesthetic inducers.

## Introduction

Voice-induced synesthesia is a relatively rare type of synesthesia. According to a database compiled by Simner and Ward using an extensive questionnaire (https://www.survey.bris.ac.uk/sussex/syn), less than 10% of the synesthetes filling out the form have voice-induced synesthesia. In this variant, people experience synesthetic perceptions induced by the sound of people's voices. Aside from one recent case study (Fernay et al., 2012), there has been no systematic research into this form of synesthesia, and no group study has been reported. According to personal reports by synesthetes from our participant pool, the most common synesthetic perceptions (so-called “concurrents”) that accompany the sound of voices are colors, textures, shapes and movements/spatial arrangements. Informal reports from approximately 15 voice synesthetes, which have been gathered from both personal communication and via an international synesthesia email forum (http://www.daysyn.com/Synesthesia-List.html) illustrate the condition's multiple facets and complexity. For example, some voice synesthetes “see” the voice better when the person is singing. For some synesthetes colors vary little between voices but for others, colors depend strongly on the individual speaking. Concurrents may also be influenced by familiarity with the voice or the medium it is transmitted through, such as direct personal communication vs. radio. Some voice synesthetes identify the pitch to be a strong influence whereas others cannot define any criteria of the voice that change their concurrents. In Fernay et al. (2012), the synesthete's perceptions included color, size, and location of the associations. The authors found that a higher pitch, or fundamental frequency (f0), resulted in lighter color associations and a higher position in vertical space. Male voices induced larger shapes than female voices.

Coming to grips with voice-induced synesthesia requires a critical analysis of the concept of “a voice.” The voice of a speaker can be distinguished from the linguistic information that the voice carries when talking (i.e., vowels, consonants, words, and intonation patterns), even though these types of information are always intertwined in the acoustic speech signal. The voices of individual speakers differ for a number of reasons, including the anatomy and physiology of the speech organs, and aspects of learned behavior reflecting group affiliation (Esling, 1978; Stuart-Smith, 1999) as well as individual idiosyncrasy and habit. Variation among voices is complex yet principled, and the approach taken in this paper is to find out what light can be cast on voice synesthesia by a systematic phonetic analysis of voices.

The phonetic concept of *voice quality* (Abercrombie, 1967; Laver, 1980) is a specialist term describing characteristics “which are present more or less all the time a person is talking: [voice quality] is a quasi-permanent quality running through all the sound that issues from his mouth” (Abercrombie, 1967, 91). The most widely-adopted framework for the analysis of voice quality is the auditory componential analysis pioneered by Laver (1980) and further developed by Nolan (1983) and Beck (2005) which analyses the voice in terms of long-term settings of the various speech organs, most importantly the larynx, the vocal folds, the soft palate, tongue, lips and jaw. *Laryngeal* settings relate to the position of the larynx in the neck, and to the mode of vibration of the vocal folds: a number of modes are distinguishable, including not only full periodic (modal) vibration of the folds, but falsetto voice (with taut vocal folds), creaky voice (with slow, irregular vibration of the folds), breathy voice (with high airflow), whispery voice (with audible friction produced by incomplete closure of the vocal folds), and harsh voice (resulting from constriction of the ventricular folds). *Supralaryngeal* settings include the location of the center of gravity of the tongue body, the degree of raising or lowering of the soft palate (which affects the degree of air escape through the nose, giving rise to the contrast between a “stuffed-up,” denasal voice, and a nasal twang), and the positions of the lips (spread, protruded) and jaw (raised, lowered, protruded).

In addition to voice quality, speakers are characterized by the pitch of their voices, or more technically by f0 (the fundamental frequency, determined by the rate of vocal fold vibration). The limits on the range of f0 that a speaker can produce are determined anatomically and physiologically (larger vocal folds produce lower frequencies) but every speaker is able to produce extensive variation in f0, the so-called pitch range. The size of the supralaryngeal vocal tract also affects its natural resonances, known as *formants*, with larger vocal tracts having lower resonant frequencies. Within the range determined for each speaker by their anatomy and their supralaryngeal settings, formant frequencies vary constantly during talking as the configuration of the vocal organs is changed to produce a sequence of vowels and consonants.

So how might these voice qualities be brought to bear when synesthetes experience colored voices? Due to the lack of literature on voice-induced synesthesia, related studies on synesthesias induced by speech sounds or the timbre of instruments might serve as a guideline for our research questions. In music-color synesthesia, Ward et al. (2006) found that higher notes triggered the experience of lighter colors, and lower notes triggered darker colors. Additionally, and in closer relation to voice quality, they found that timbre affected lightness choice: piano and string notes triggered more “colorful” experiences (i.e., higher chroma colors) than pure tones. Both these findings suggest that voice pitch or quality may influence the colors experienced by voice-triggered synesthetes. In linguistic synesthesias (e.g., triggered by spoken words), the acoustic and articulatory characteristics of vowels have also been shown to systematically influence color and luminance associations (Jakobson, 1962; Marks, 1974, 1975; Moos, 2013). Moos (2013) showed that acoustic measures (formant measures) could be used to analyse and explain inducer-concurrent relations.

In addition to color, voice-triggered synesthetes often report texture perceptions, for example a voice might be “smooth but granulated” or “[with a] soft center and very slight fuzziness around the outside.” Despite our knowledge of perceptions such as these, no systematic investigation of visual texture perceptions in synesthesia has yet been conducted—which is perhaps not surprising considering that it is not easy to quantify texture or to relate this quantification to perceptual categories (Petrou et al., 2007; Clarke et al., 2011). Eagleman and Goodale (2009) state: “Quantitatively testing these prevalences [of texture concurrents] will be a challenge: it is straightforward to develop a user-friendly color chooser […], but not so with the multidimensional varieties of texture” (Eagleman and Goodale, 2009, 291). In the current study we take on this challenge by analysing not only color associations, but also texture.

In this first group study on voice-induced synesthesia, we set out to answer three questions. First, we assess how voice-induced synesthesia expresses itself in individuals and across groups. Specifically, we ask about precise relationships between acoustic characteristics of the voice and its synesthetic concurrents, focusing on both color and texture. Second, we test the consistency of color and texture associations over time. The consistency with which synesthetic concurrents are described by synesthetes in different test sessions over time is often taken as the hallmark of synesthesia (e.g., Rich et al., 2005), although levels of consistency have since been shown to vary according to the particular type of synesthesia under investigation (e.g., Simner et al., 2011), and the particular methodology used (e.g., Simner and Ludwig, 2012). Finally, we ask how synesthetic colors for voice might differ from the normal cross-modal associations made by the general population. This will allow us to investigate common aspects of cross-modal perception as for example discussed in Spence (2011).

In our study, we tested voice synesthetes, professional phoneticians, and control participants, conducting the experiment online to facilitate participation for people with this rare type of synesthesia. Phoneticians were included to examine the potential influence of this profession on cross-modal associations with voices. Participants heard auditory samples which were controlled recordings of (non-participant) phoneticians producing different voice qualities (see section Voice Stimuli), and which were both perceptually and acoustically clearly distinct from each other. Participants cross-modally matched these to items from a closed set of colors and textures. Additionally, free verbal descriptions were elicited to gain a richer picture of participants' associations with the voices. After 2–8 months, a retest was conducted with a subset of the stimuli to test for consistency in participants' associations. The study was approved by the ethics board of the University of Glasgow, and participants provided informed consent before testing.

## Methods and materials

### Participants

There were three groups of participants: synesthetes, phoneticians, and controls. All participants were native speakers of English and had no severe sight or hearing difficulties. Participants were paid with Amazon vouchers.

We tested 14 voice-induced synesthetes (mean age = 34, age range = 18–70, *SD* = 19; 11 female), recruited from the Sussex-Edinburgh Database of Synesthete Participants, or via announcements on an online synesthesia forum (http://www.daysyn.com/Synesthesia-List.html). Synesthete participants were initially identified by self-report and in nine cases additionally by a synesthesia questionnaire designed by Simner and Ward (https://www.survey.bris.ac.uk/sussex/syn). Genuineness is also usually confirmed with consistency tests (consistently perceiving the same concurrents for the same inducers over time); this matter will be returned to in the discussion section. Thirteen synesthetes additionally self-reported color and/or texture perceptions induced by stimuli other than the sound of voices (e.g., digits, music). On average, they had 10 different inducers, ranging from 4 to 18. Five synesthetes were students; the rest came from a variety of professional backgrounds.

We also tested 10 phoneticians (mean age = 40, age range = 24–68, *SD* = 15.9; 7 female), recruited through an announcement on a phoneticians' email list and by individually contacting colleagues outside our universities by email. Three of them were PhD students, the rest were professionals. Phoneticians were identified as being non-synesthetes using a short questionnaire describing the phenomenon of synesthesia. In this, they were shown a list of 20 possible inducers (e.g., sounds, letters, words) and asked whether any triggered spontaneous colors, textures, or other sensations. They were classified as non-synesthetes when none of the inducers were selected. Finally, we additionally tested 28 control participants (mean age = 23, age range = 18–30, *SD* = 3.5; 17 female), recruited through the participant pool at the School of Psychology, University of Glasgow. Twenty-four participants were students and the rest were professionals. The same procedure as for the phoneticians identified them as non-synesthetes.

### Materials

Our materials comprised a set of voice stimuli, and a response display showing a set of colors and a set of textures. (The response display also contained a set of semantic differentials which were included for another study presented elsewhere; Moos, 2013; Moos et al., in press). These are described in turn below.

#### Voice stimuli

Materials were two short spoken passages taken from the story “The rainbow passage” (Fairbanks, 1960): “These take the shape of a long round arch, with its path high above, and its two ends apparently beyond the horizon” and “People look, but no-one ever finds it.” To avoid the influence of color terms on participants' perceptions, these sentences did not contain any color information. Our materials were recorded by two male phoneticians, who were able to deliberately vary their voice quality settings. We thereby avoided using many different speakers among whom voice quality would vary in less constrained ways. Ten voice qualities were chosen based on the criterion that they were perceptually maximally different. These were as follows:

**Table:** 

*MODAL*	neutral setting of speech organs; sound of a healthy voice
*NASAL*	additional air flow through the nose
*DENASAL*	no air flow through the nose, as if the nose was blocked
*RAISED LARYNX*	with an elevated larynx, sounding slightly strained and higher pitched
*LOWERED LARYNX*	with a lowered larynx, sounding slightly relaxed and lower pitched
*WHISPER*	no voicing, turbulent airflow only
*FALSETTO*	so called “head voice,” high pitched with taut vocal folds
*HARSH*	tense and rough irregular voicing, with constriction of the ventricular folds
*BREATHY*	soft and lax voice with an increased air flow due to incomplete closure of the vocal folds
*CREAK*	low pitched irregular voicing with slow, irregular vibration of the vocal folds.

To facilitate online access, recordings were converted from 11 kHz wave files into mp3 format with a bit rate of 192 kbps. The intensity was equalized for all sound files to 70 dB_SPL_ using Praat (Boersma and Weenink, 2012) to avoid differences in volume. With two speakers, two sentences and ten voice qualities, there were 40 stimuli in total. One sound sample per voice quality can be found online in the supplementary material.

In preparation for our quantitative analysis, the voice recordings were also acoustically analyzed using Praat (Boersma and Weenink, 2012) or WaveSurfer (Sjölander and Beskow, 2005). To reduce the amount of data for treatment in our main experiment, we fed a set of 14 possible acoustic features into a factor analysis. Features with strongly correlating scores were reduced into one group, which we named according to whichever feature had the strongest regression coefficient within it. The resultant four features are defined below, and their quantitative values are given in Table 1 (which shows their values across each of the ten different voice qualities, averaged from the two speakers and across the two sentences).

**Table:** 

*f0*	Mean fundamental frequency of the voice recording; this relates to the overall pitch of the voice.
*LTF2*	Long-term formant distribution (LTF) of the second vowel formant^*^ frequency (F2). As an average of all vowels in the recording, LTF2 conveys information about general vocal tract settings and vocal tract size. See Moos (2010) and Nolan and Grigoras (2005) for more details on using LTF.
*Spectral tilt*	Energy distribution across the frequency range measured in one accented vowel per recording (“app*a*rently” for sentence 1, “*e*ver” for sentence 2). Spectral tilt is the extent to which energy in the signal falls off as frequency increases: energy at higher frequencies is less damped when spectral tilt is shallow and more damped when spectral tilt is steep. It relates to various physiological characteristics of vocal fold vibration, including the proportion of the vibratory cycle during which the folds are open, and the abruptness or gradualness of vocal fold closure. BREATHY voice, e.g., is associated with a steep spectral tilt, CREAK and HARSH among others with a shallow tilt. Spectral tilt was quantified here using measures of the corrected first harmonic minus the corrected amplitude of F3 (H1^*^-A3^*^) (see Hanson, 1997 for further details).
*Pitch range*	Variability of f0 in a speaker, calculated by subtracting the minimum from the maximum pitch of a voice recording and converting to semitones. This describes the differences between (for example) a “singsongy” vs. monotonous voice (i.e., large vs. small pitch range).

* Formants are spectral peaks of intensity at different frequencies (usually measured in Hz) in the frequency spectrum of the sound. They are created by the resonances of the vocal tract (Clark et al., 2007). A vowel sound contains several formants. The lowest two formants mainly characterize the vowel quality, while all formants additionally give information about speaker characteristics.

**Table 1:** **Acoustic values for the different voice qualities, averaging across speakers, and sentences**.

**Voice quality**	**f0 (Hz)**	**LTF2 (Hz)**	Spectral tilt H1^*^-A3^*^ (dB)	**Pitch range (semitones)**
MODAL	119	1339	20.32	12.22
RAISED LARYNX	156	1245	13.19	13.68
LOWERED LARYNX	124	1247	19.83	8.89
NASAL	110	1352	19.49	9.30
DENASAL	114	1341	17.63	9.14
FALSETTO	232	N/A	N/A	10.24
BREATHY	109	1319	23.33	7.46
WHISPER	N/A	1463	0.30	N/A
HARSH	106	1408	−0.52	6.42
CREAK	92	1311	14.96	9.47

Pitch-related values could not be entered for WHISPER because there is no voicing in WHISPER. Formant-related measures cannot be given for FALSETTO because the pitch is so high that formant frequencies cannot be accurately resolved.

#### Response display

***Colors.*** A forced choice response display was presented with 16 different colors, comprising the 11 focal colors of English (Kay et al., 2009): white, black, blue, green, yellow, red, gray, brown, orange, pink, and purple, plus an additional five colors also varying in luminance: pale pink, dark green, light green, cyan and dark blue. This limited set was preferred over an unlimited color picker to reduce task demands and shorten the time required of our participants. Participants also had the opportunity to describe fine-grained details of their color associations in a verbal response (see below).

Our color stimuli were created by entering red, green, blue (RGB) values into our computer display and these colors were subsequently quantified for our main analysis using a Minolta CS-100 chromameter and converted into CIELUV color space (Westland and Ripamonti, 2004). The chromameter measured the luminance (L) and chromaticity (x, y) values on ten different computer screens in different lighting conditions to get an estimate of the variation of settings that participants would use. The average of these ten measures was then used to convert the numbers into co-ordinates within the CIELUV color space, using the formula published in Westland and Ripamonti (2004, p. 50f). This color space is suitable for self-luminous colors such as those displayed on computer screens, and achieves perceptual uniformity (i.e., a given change in color value produces the same visual significance regardless of where in color space that change occurs). Within this color space, colors are represented by L^*^, u^*^, and v^*^ co-ordinates, representing luminance, red-green and yellow-blue respectively. When converting our colors, our “reference white” was taken from the background gray, to place our palette in the correct color context. This occasionally resulted in L^*^ values above the usual upper limit (100) when white is used as reference. Both RGB and L^*^, u^*^, v^*^ values are listed in Table 2. A positive u^*^ value stands for red tint and a negative one for green tint; a positive v^*^ value stands for yellow tint and a negative one for blue tint. A high L^*^ value stands for light and a low one for dark.

**Table 2:** **RGB values and CIELUV coordinates of the 16 colors used for creating the color patches in the online survey**.

**Color**	**R**	**G**	**B**	L^*^	u^*^	v^*^
White	255	255	255	129.8	−1.6	6
Yellow	255	225	0	121.9	30.8	125.5
Cyan	0	220	220	110	−80.6	−23
Pale pink	255	175	175	109.4	44.3	4.4
Olive	150	200	0	105.4	−29.2	111.5
Orange	255	128	0	92.6	124.6	83.2
Green	0	160	0	82.1	−67.5	88.9
Pink	255	0	255	81.3	92	−124.2
Grey	115	115	115	74.4	−5.4	−6.3
Red	255	0	25	73.9	206	52.4
Blue	0	100	255	69.6	−39.8	−154.9
Purple	120	0	150	48.7	32.5	−107.4
Brown	110	60	0	48.1	51.7	38.4
Dark green	0	75	0	45.1	−30.3	42.5
Dark blue	0	50	128	41.5	−19.9	−95.1

L^*^, u^*^, v^*^ values calculated from the average L, x, y measures of ten randomly selected computer screens.

**Figure 1:**
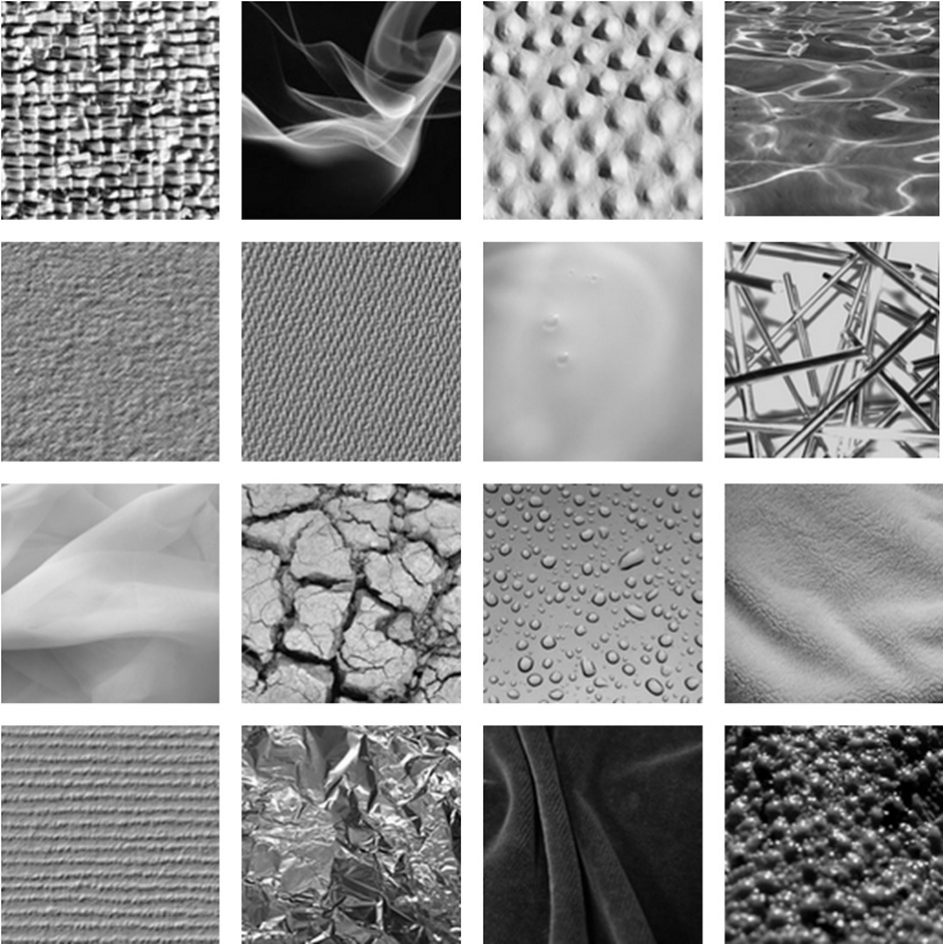
**Sixteen textures used in the response display**.

***Textures.*** Our response display also presented visual representations of 16 textures (Figure 1). The selection of textures was dictated by those mentioned most often in synesthetes' descriptions of their textural concurrents, communicated in forum posts in a synesthesia community (http://www.daysyn.com/Synesthesia-List.html) and through personal communication. The textures most often named were: rough, liquid/fluid, smooth, shiny, hard, dry, soft, bumpy, sharp, bubbly, milky, transparent, metallic, and textiles like velvet, linen, flannel, corduroy, plaid, and felt. For logistical reasons this list of texture descriptions was reduced to 16 for use in the experiment, with each texture designed to be close to the descriptive words used by the synesthetes, but distinct from the other textures. The textures in Figure 1, from left to right and top to bottom, are referred to as: 1. rough, 2. smoke, 3. bumpy, 4. water, 5. rough-ish, 6. jeans, 7. milk, 8. sharp, 9. net, 10. dry, 11. drops, 12. fleece, 13. stripes, 14. foil, 15. velvet, and 16. bubbly. Textures were uniform with respect to their simulated viewing angle, and presented as gray-scale images to avoid a confounding influence of color. Pictures were taken from the database created by Halley and colleagues (Clarke et al., 2011; Halley, 2011), Brodatz (1966) and from homepages without copyright limitations.

Our limited set was selected to allow for an assessable display, and to make the data manageable for analysis. To prepare for our analysis, the textures were quantified using human ratings gathered from 32 native English speaking participants (9 female, mean age = 26, *SD* = 8 years) who did not take part in the main experiment. Human ratings were selected since these match the perceptual space of textures better than computer algorithms (Clarke et al., 2011). Participants rated each texture along eight semantic scales following textural classifications in Rao and Lohse (1993, 1996) and Tamura et al. (1978), presented on horizontal sliders with descriptive words at opposing ends, as follows:

- rough—smooth
- fine—coarse
- low contrast—high contrast
- high complexity—low complexity
- repetitive—non-repetitive
- non-directional—directional
- line-like—blob-like
- regular—irregular

Participants' ratings were fed into a factor analysis to again reduce the amount of data for treatment in our main experiment. Semantic differentials whose scores were strongly correlated with a common latent variable or underlying dimension were reduced into one group, i.e., a factor. Each factor was named according to the semantic differential that had the strongest regression coefficient with it: repetitiveness, roughness, complexity, and line- vs. blob-likeness. Rating results of these four semantic differentials for each individual texture image can be viewed in the supplementary material. Factor loadings, sums of squares and variance data are given in Table 3.

**Table 3:** **Factor analysis with promax rotation of texture rating data**.

	**Factor 1 repetitiveness**	**Factor 2 roughness**	**Factor 3 complexity**	**Factor 4 line- vs. blob-like**
Rough—smooth		***0.962***		
Fine—coarse		**−0.952**		
Low—high contrast	−0.154		**0.862**	
High—low complexity	−0.183		***−0.877***	
Repetitive—non-repetitive	***1.015***	0.123		−0.214
Non-directional—directional	**−0.791**	0.130		−0.390
Line-like—blob-like				***0.998***
Regular—irregular	**0.906**		0.204	
SS loadings	2.542	1.874	1.562	1.208
Proportion variance	0.318	0.234	0.195	0.151
Cumulative variance	0.318	0.552	0.747	0.898

Top: rotated factor loadings of the semantic differentials for four factors. Bottom: sums of squares (SS) loadings of the above factor loadings and the proportional and cumulative variance of the data that they account for. Bold typeface indicates a strong regression coefficient of the semantic differential with a factor. The semantic differential with the strongest regression coefficient, chosen as representative of a factor, is indicated in italics.

In summary, our materials comprised 40 voice samples (two speakers × 10 voice qualities × two sentences), as well as a response palette showing 16 colors (each quantified by its L^*^, u^*^, v^*^ values) and 16 textures (each quantified along eight semantic scales).

### Procedure

The experiment was conducted online using the software LimeSurvey (www.limesurvey.org). Participants were encouraged to use the best possible audio equipment at their disposal, usually headphones or external speakers.

Participants heard each audio file one at a time (and could replay each as often as needed before advancing) and were asked the following question: “What are your first impressions of and associations with this voice? Please describe the voice in your own words.” Participants entered their replies in a text box. Below the box, there were eight sliders with the semantic differentials (the results of which are presented elsewhere; Moos, 2013; Moos et al., in press). This was followed by the color display on the same screen with the question “Which color matches the voice best?” and the texture display asking participants to choose the best match as well. At the bottom of the screen, there was space for optional comments: “On a scale from 0 to 9 (where 0 is nothing and 9 very intense), how intense are your color and texture experiences? Is there anything more you want to add?” Every time a stimulus was accessed on the homepage, the semantic differentials were displayed in random order, as were the color and the texture display.

Our materials were presented in a block design in which block 1 was the 20 recordings of sentence 1 (10 voice qualities × 2 speakers), followed by a screen that collected demographic data (where participants had the option to save results and return at a later point), followed by block 2, which was the 20 recordings of sentence 2 (again, 10 voice qualities × 2 speakers). Within blocks, all trials were presented in a random order. The study ended with a voice comparison task (for a study reported elsewhere) and a short synesthesia questionnaire collecting data about participants' types of synesthesia. The experiment lasted for about 1.5–2 h. After 2–8 months, a retest was conducted with a subset of the stimuli to test for consistency in participants' associations (in all but three cases, after 5–8 months). The subset comprised each voice quality once, with five voice qualities produced by speaker 1 and five by speaker 2. For five stimuli, sentence 1 was used, for the other five, sentence 2. This resulted in 10 stimuli. Twelve synesthetes, 10 phoneticians, and 20 controls took part in the retest.

## Results

We first consider the cross-modal associations of our participants as they were given in the initial testing session. Here we analyzed participants' responses from both their qualitative/verbal descriptions, and their responses given via our color/texture response-display. Subsequent to this, we analyzed their consistency over time by comparing responses in the first vs. second testing sessions. We present these analyses in turn.

### Qualitative results of verbal descriptions

Participants' verbal descriptions of associations to our voice stimuli were coded based on the systematic methodology of the Grounded Theory (Strauss and Corbin, 1998), and each reply was inspected at least twice during coding. In total, 25 codes were created which were subsequently grouped into six different categories as listed below:

**Table:** 

1. Associations	
*Person*	associations with real or fictitious people
*Anno*	time period of recording
*Pre*	comparing the speaker to one previously heard
2. Description of person	
*Age*	age
*Sex*	sex of speaker
*Occupation*	occupation
*Look*	physical appearance, clothing
*Health*	state of health
*Personality*	character, habits, attitudes
*Emotions*	emotional state of the speaker, feelings of the speaker
3. Feelings in listener	
*Feelings*	emotions or feelings evoked in the listener
4. Phonetics	
*Voice quality*	professional terminology or layperson's description of voice quality
*Phonetics*	other terminology related to phonetics (other than relating to voice qualities), e.g., pitch or speaking rate
*Accent*	regional area, accent
*Fake*	disguised or pretend voice
*Evaluation*	evaluation of the voice, e.g., “good voice,” “could be better if … ”
*Style*	speaking style, e.g., “story telling,” “newsreader,” “telling off…”
5. Synesthetic perceptions	
*Color*	color terms
*Texture*	texture terms
*Shape*	terms describing a shape
*Space, movement*	terms describing where in space something is positioned and/or whether it moves
*Taste*	gustatory terms
*Smell*	olfactory terms
*Temperature*	terms related to temperature
6. Unclassified	
*Misc*	terms not falling within the categories above.

We tested whether the use of verbal descriptions differed between the three subject groups using a MANOVA, with Gabriel's test for *post-hoc* tests because group sizes were different. The dependent variable was defined as the number of times a category was assigned to the verbal descriptions per participant. Testing the difference between the six categories produced four significant main effects, of: Phonetics [*F*_(2)_ = 14.90, *p* < 0.001], Description of person [*F*_(2)_ = 4.08, *p* = 0.023], Feelings in listener [*F*_(2)_ = 4.69, *p* = 0.014], and Synesthetic perceptions [*F*_(2)_ = 22.8, *p* < 0.001]. Planned comparisons explain these differences as follows. As would be expected, phoneticians used phonetic descriptions on average 35.2% more than synesthetes [*t*_(1)_ = 5.16, *p* < 0.001]. Synesthetes used synesthetic descriptions on average 38.7% more than phoneticians [*t*_(1)_ = 5.15, *p* < 0.001] and 38.2% more than controls [*t*_(1)_ = 6.42, *p* < 0.001]. Perhaps reflecting their lower use of synesthetic descriptions, controls instead used descriptions of the speaker on average 13.5% more than synesthetes [*t*_(1)_ = 2.53, *p* = 0.039]. They also used phonetic descriptions 22.9% more than synesthetes on average [*t*_(1)_ = 4.24, *p* < 0.001]. Finally, synesthetes also described their feelings on average 4.1% more often than phoneticians [*t*_(1)_ = 2.87, *p* = 0.017], and this perhaps reflects the affective quality of synesthesia (Callejas et al., 2007).

**Figure 2:**
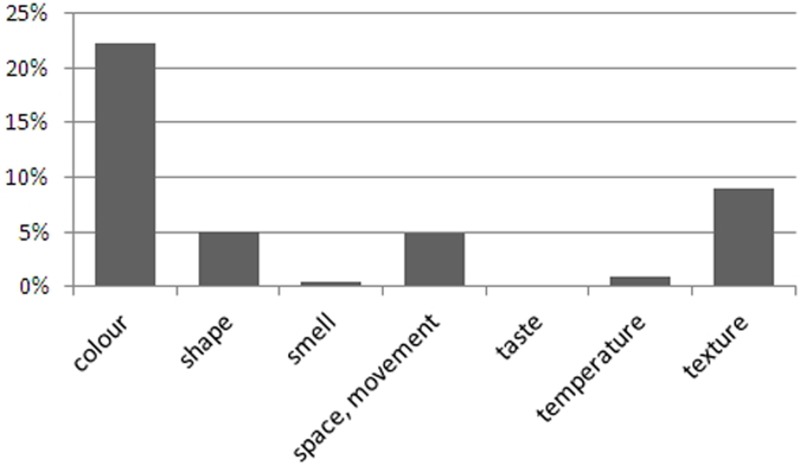
**Use of synesthetic codes for verbal descriptions by synesthetes.** The result for taste is 0.1%.

The data also show that synesthetes used terms from the “synesthetic perceptions” category in 42.7% of their responses, although the range was rather wide (2–94%). Figure 2 breaks down these responses to show the types of concurrent modalities expressed. This finding confirms previous research that most concurrents are colors (Day, 2005; Novich et al., 2011). It also supports our decision to use color and texture displays as these are the most frequent concurrents. However, care must be taken since synesthetes' verbal descriptions may not indicate their range of concurrents, *per se*, but rather, the different degrees to which they might express them in this task.

### Response display

#### Color associations

One way to consider analysing color responses is by color category (e.g., FALSETTO voice might generate more “pink” responses, and CREAK voice might generate more “gray” responses). This approach to analysing voice quality is presented in Moos et al. (in press). Here we instead consider how quantified measures of different voice qualities map onto the quantified measures of color (and below, texture).

The influence of the acoustic measures of the voice qualities on the CIELUV coordinates of the associated colors was tested using linear mixed effects modeling carried out in R (Baayen, 2008). L^*^, u^*^ and v^*^, representing luminance, red-green and yellow-blue, respectively, were used as dependent variables. Our four acoustic features, namely f0, LTF2, spectral tilt and pitch range, were used as predictors, as was participant group, and the interactions of group with each acoustic feature. Participants were included as random effects. Table 4 shows our significant results, as non-significant predictors were not retained in the model.

**Table 4:** **Results of linear mixed effects modeling testing acoustic influences on participants' color associations**.

		***t***	***p***	**Qualitative explanation**
L^*^	f0 mean	12.57	<0.001	Higher f0, lighter
	pitch range	6.58	<0.001	Larger range, lighter
	LTF2	2.46	0.014	Higher LTF2, lighter
	group (control vs. phoneticians)	−3.52	<0.001	Phoneticians darker
	LTF2^*^group (control vs. phoneticians)	3.67	<0.001	Phoneticians > control
u^*^	f0 mean	5.30	<0.001	Higher f0, redder
v^*^	spectral tilt	−2.31	0.021	Steeper tilt, bluer
	pitch range	2.50	0.012	Smaller range, bluer

In summary, our results were:

- A higher f0 (as in FALSETTO), a higher LTF2 (as in WHISPER) and a larger pitch range (as in RAISED LARYNX) led to lighter color choices across groups, whereas a lower f0 (as in CREAK), a lower LTF2 (as in LOWERED LARYNX) and a smaller pitch range (as in HARSH) resulted in darker color associations.
- A higher f0 led to redder color choices across groups, whereas a lower f0 resulted in greener color associations.
- A steeper spectral tilt (as in MODAL voice) and a smaller pitch range led to bluer color choices across groups, whereas a shallower spectral tilt (as in RAISED LARYNX) and a larger pitch range resulted in yellower color associations.

Color associations with FALSETTO, for example, illustrate the dominance of red-related associations with high f0, as a majority of participants chose red, pink, pale pink, purple and orange, resulting in high u^*^ values. The lower luminance values for color associations with CREAK, for example, result from black, brown, dark blue and gray selections by a large part of the participants.

Table 4 also reveals a group difference between controls and phoneticians on the luminance scale: phoneticians associated the voices with significantly darker colors than controls. LTF2 interacts with group in the following way: phoneticians use the luminance scale more extensively in response to changes in LTF2 than controls do; there is no significant difference between synesthetes and others.

#### Texture associations

First, a visual impression of associations between voice quality and texture is given in Table 5. It lists the four voice qualities that evoked the highest agreement in terms of texture associations both within and across groups, i.e., those associations shared by most participants. With 16 textures offered in the response display, chance level of associations between the different textures and a voice quality is 6.25%. The strongest agreement was found for the association of the smoke-like texture with WHISPER. This texture image may have evoked thoughts of high-frequency noise travelling through the dark; some participants described associations with darkness for WHISPER because the darkness of the night is a common environment for whispering. The associations of dry cracked soil with HARSH and CREAK seems intuitive as these voice qualities give an auditory impression of a dry throat.

**Table 5:** **The four voice quality—texture associations with highest agreement between and across groups**.

**Textures**	**Voice qualities**	**Total**	**Synesthetes**	**Phoneticians**	**Controls**
		**in %**			
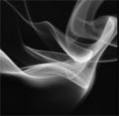	WHISPER	30	36	30	29
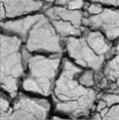	HARSH	26	36	30	22
	CREAK	20	25	18	20
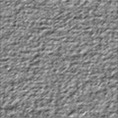	NASAL	17	18	15	18

Linear mixed effects modeling was used to assess the influence of voice acoustics on texture associations and to reveal potential group differences in those associations. The four semantic qualities “rough—smooth,” “high—low complexity,” “repetitive—non-repetitive” and “line-like vs. blob-like” were used as dependent variables, representing the texture choices of participants. The four acoustic features were again used as predictors. Detailed significant statistical results, with brief qualitative explanations in the last column, are shown in Table 6. Again, non-significant predictors were not retained in the model.

**Table 6:** **Results of linear mixed effects modeling testing acoustic influences on participants' texture associations**.

		***t***	***p***	**Qualitative explanation**
Rough—smooth	f0 mean	2.63	0.009	Higher f0, smoother
	Pitch range	2.30	0.022	Larger range, smoother
	LTF2	4.30	<0.001	Higher LTF2, smoother
	Spectral tilt	5.18	<0.001	Steeper tilt, smoother
High complexity—low complexity	f0 mean	−3.02	0.003	Higher f0, more complex
	Pitch range	4.33	<0.001	Smaller range, more complex
	LTF2	−2.13	0.034	Higher LTF2, more complex
	Spectral tilt	7.11	<0.001	Shallower tilt, more complex
	Group (syn vs. con)	−2.40	0.016	Synesthetes more complex
	Group (syn vs. phon)	−2.46	0.014	Synesthetes more complex
Repetitive—non-repetitive	f0 mean	4.40	<0.001	Higher f0, less repetitive
	Pitch range	−2.48	0.013	Smaller range, less repetitive
	LTF2	5.93	<0.001	Higher LTF2, less repetitive
Line-like—blob-like	Spectral tilt	3.61	<0.001	Steeper tilt, more blob-like

It was found that both higher pitch and higher LTF2 were associated with textures that were “smoother,” “more complex,” and “less repetitive.” A steeper spectral tilt as in BREATHY resulted in “smoother,” “less complex,” and “blob-like” texture choices, whereas a larger pitch range as in RAISED LARYNX triggered choices of textures with “more repetitive” patterns. Key results are summarized in Table 7. A small but significant group difference in the usage of textures was found for the complexity scale: synesthetes chose “more complex” textures than phoneticians and controls. This could be due to the fact that synesthetic concurrents are on average more complex in their structure than associations of non-synesthetes which are only consciously present when triggered by visual input.

**Table 7:** **Summary of significant influences of acoustic characteristics of the voices on the associated textures**.

**Acoustic characteristics**		**Associated texture characteristics**		
Pitch (f0)	High	Smoother	More complex	Less repetitive
	Low	Rougher	Less complex	More repetitive
Pitch range	Small	Rougher	More complex	Less repetitive
	Large	Smoother	Less complex	More repetitive
LTF2	High	Smoother	More complex	Less repetitive
	Low	Rougher	Less complex	More repetitive
Spectral tilt	Steep	Smoother	More complex	Blob-like
	Shallow	Rougher	Less complex	Line-like

### Retest

In the retest, synesthetes chose exactly the same color again 25% of the time, phoneticians 21% of the time and controls 15% of the time. Synesthetes chose exactly the same texture again 15% of the time, phoneticians 19% and controls 13% of the time (see Moos et al., in press; Moos, 2013 for more details). Synesthetes' consistency scores ranged from 10 to 60%. Although there is a tendency for synesthetes to outperform controls in consistency, the scores do not reach levels of consistency found, for example, in grapheme-color synesthesia, which are usually above 70% (Asher et al., 2006; Simner et al., 2006). Two reasons may explain this result. First, the complexity of voice as an inducer (combination of voice quality, intonation, content of words etc.) plus the additional types of synesthesia of the participants suggest that results cannot be compared to a relatively clear-cut and well-researched type such as grapheme-color synesthesia. Second, the visual response displays may not have resembled the exact synesthetic reactions. To circumvent this issue, we also tested whether participants chose colors and textures *similar* to their choices in the initial test.

For this, a MANOVA was conducted. Tukey's method is reported for the *post-hoc* test because this is powerful while having good control over the Type I error, and results differed little from those of Gabriel's method which takes into account the different group sizes. L^*^, u^*^ and v^*^ were used as the measures for color. Testing consistency across voice qualities, all group differences for color associations were non-significant [*F*_(2)_ = 2.16, *p* = 0.117 for L^*^; *F*_(2)_ = 2.07, *p* = 0.127 for u^*^; *F*_(2)_ = 2.56, *p* = 0.079 for v^*^]. However, v^*^ approached significance between synesthetes and controls (*t* = 12.37, *p* = 0.063), indicating that synesthetes are marginally more consistent in the yellow-blue dimension of their color associations.

Interestingly, results for texture associations across voice qualities show partly higher consistency for phoneticians than synesthetes: phoneticians were more consistent in choosing “rough-smooth” (*t* = 6.40, *p* = 0.04) and “fine-coarse” textures (*t* = 4.50, *p* = 0.053). It could be argued that the (ir)regularity of vocal fold vibration has a strong textural parallel. Harsh and creaky voice qualities are examples of irregular voicing, with harsh often being called rough in lay terms. Also, visual textural patterns emerge from the phonetician's tool for speech analysis, the spectrogram, which looks smoother for a modal voice and coarser for a harsh or creaky voice. Phoneticians may therefore be most consistent in these measures.

## Discussion

### Main findings

We conducted an exploratory study on voice-induced synesthesia using both qualitative and quantitative analyses. The qualitative approach—coding and analysing the verbal descriptions—gave insights into the different ways participants perceive and express their perceptions of different voice qualities. This was a necessary first step toward understanding this under-researched type of synesthesia. As conjectured, synesthetes regularly used their synesthetic perceptions to describe voices, mostly color and texture terms; phoneticians used more technical terms; controls focussed on describing personal characteristics of the speaker. Nevertheless, individual differences in the use of synesthetic descriptions and in the consistency range of color and texture associations were found within the group of synesthetes. The use of synesthetic terms in the verbal descriptions ranged from 2 to 94% across individuals, while the range of test-retest consistency in choosing identical colors and textures for the same voice qualities varied between 10 and 60%.

Parameterization of the color and texture choices made it possible to quantify how participants' responses were influenced by acoustic attributes of the different voice qualities. Higher fundamental frequencies were associated with lighter colors across groups. This is in line with the findings of Ward et al. (2006) on musical pitch, whereas de Thornley Head (2006) found group differences: pitch changes did not affect the lightness of his synesthetes' color choices, but did influence control participants. A recent study on pitch-luminance mapping found that even chimpanzees prefer to match white to high pitched sounds and black to low pitched sounds (Ludwig et al., 2011). This finding suggests a common underlying mechanism of sensory processing in primates, which seems to be hard-wired rather than acquired through culture or language. Neurons in the auditory cortex are organized tonotopically according to the frequency of sound to which they respond best in both humans and other primates (Lauter et al., 1985; Talavage et al., 2004; Bendor and Wang, 2005), similar to the arrangement of sound processing in the cochlea. This frequency map in the auditory cortex may possibly relate systematically to a luminance map in V4.

Other acoustic effects on responses are less easy to explain in terms of frequency-luminance matching and may be mediated by connotative influences. A higher f0 also led to redder color choices in our participants, e.g., for falsetto, where red, pink, pale pink, purple, and orange associations dominated the responses. Although not statistically demonstrated by de Thornley Head (2006), the graphs on p. 170 suggest that his participants also associated redder colors with higher pitched tones. A larger pitch range (as in raised larynx) resulted in lighter and yellower color choices across groups, while a steeper spectral tilt (as in breathy) triggered more blue associations across groups. Some voice qualities are judged to have different degrees of pleasantness. A breathy or modal voice, for example, is usually perceived as pleasant to listen to (Reich and Lerman, 1978). In our study a high f0, high vowel formants, a larger pitch range and a steeper spectral tilt resulted in associations with textures that were “fluid,” “smooth” and have, according to Reich and Lerman (1978) and Lucassen et al. (2011), a pleasant connotation, whereas a shallow spectral tilt (as in harsh) resulted in “rough” and “line-like” texture choices and those with unpleasant connotations.

Lucassen et al. (2011) published a study on the affective connotations of texture and color. The authors list semantic differentials for color and texture ratings, namely warm-cool (for colors only), feminine-masculine, hard-soft and light-heavy. They found that more complex textures are more masculine, hard, and heavy. Light weight is associated with light colors and heavy with dark colors. Softness is associated with less saturated colors than hardness. Femininity is rated as more pink and masculinity more blue, green and dark. Warmth is perceived as more red and brown, whereas coolness is more blue and green. Although a detailed comparison between Lucassen et al.'s experiments and ours is not possible, parallels can be found for the feminine-masculine scale and ratings for f0 by our participants: high f0 (falsetto, reaching frequencies typically associated with a female voice) was associated with redder colors than low f0, matching Lucassen et al.'s findings exactly. Furthermore, one synesthete had temperature concurrents with the voices which she expressed in her verbal descriptions. It is noticeable that her descriptions of warm temperature are often accompanied by red color choices and cold temperature by green or sometimes blue color choices. The interesting likely relationship between affective responses and color/texture associations is potentially worthy of further investigation, particularly in the light of visual aesthetics (Palmer et al., 2013).

### Methodological issues

The use in this study of visually-presented textures as opposed to tactile presentation (Simner and Ludwig, 2012) appears to have been successful. Simulated viewing angle was kept constant and textures presented in grayscale to avoid confounds with hue. A potential confound of the texture images *per se* and their luminance was ruled out *post-hoc*: Texture images that were light overall were not associated with the same voice qualities which induced light colors. The picture of the dry, cracked soil, for example, has a relatively high luminance overall; but voice qualities which were associated with this picture were generally given dark color associations.

Occasionally, some synesthetes complained about the limited set of colors. However, using a more complicated color response display was deemed cumbersome, as it would have made an already lengthy experiment even longer. The synesthetes who found the set of colors to be limited used the opportunity to detail their color perceptions in the verbal descriptions: a majority of them used customized color terms, such as “tonic green” or “dark wine color” or named more than one color to describe a voice (see Moos et al., in press, for more details). The set of textures was also not comprehensive. It is unclear how to present textural displays more optimally to synesthetes, and there is little research on this topic. One option would be a browsing environment similar to that presented by Halley (2011) and Clarke et al. (2011), although this would be very time consuming. There were fewer comments by synesthetes about the limited set of textures than about that of colors. Potentially, their synesthetic reactions were less clear or more indifferent for textures than for colors; or they found it easier to match their concurrents to the given set because their textures were more similar to those on display.

Consistency in people's associations was measured for textures and colors through a retest. Synesthetes were marginally more consistent in some of their color associations than controls, while phoneticians showed regular patterns in some measures that underline their expert knowledge of assessing voice qualities, e.g., the extensive use of the smooth-rough scale. However, the main—and unexpected—pattern to emerge from tests of consistency of synesthetic perceptions was the lack of significant differences between groups. This surprising lack of strong consistency in synesthetes' choices leaves four main interpretations.

First, it is possible that some of our group were not true voice synesthetes. Of course we tried to rule out this possibility by conducting synesthesia tests with questionnaires. Moreover, the richness of the verbal descriptions provided by the synesthetes supports the idea that they were genuine: Simner et al. (2005) have shown that verified synesthetes use significantly more descriptive color terms when describing concurrents. Nonetheless, there cannot be absolute certainty with an under-researched type of synesthesia such as voice-induced synesthesia. Second, the stimuli might have evoked voice synesthesia less strongly, and therefore less consistently, than would have been optimal. Perhaps varying voice qualities within two speakers gave in fact less perceived variation than might have been obtained using many different speakers including female voices (cf. Fernay et al., 2012), even though acoustic variation was present within the stimulus set.

Third, it might be the case that voice-induced synesthesia is not as easily defined as other types of synesthesia; ergo, the definition of synesthesia needs to be revised, especially regarding consistency as a main criterion, as has recently been suggested by Simner (2012a,b). Fourth, and relatedly, perceptions might be influenced by other types of synesthesia to a certain degree, in ways which could not be separated out in the analysis. A synesthete might attend to the voice in the first run of the test and have the corresponding synesthetic reactions. In the second run, she might attend to the words being said which induce different synesthetic reactions. The influence of other synesthesias is one of the largest difficulties faced in this experiment.

All but one of the 14 synesthetes reported having at least four other types of synesthesia. Of these, 13 had music as an additional inducer, 11 had letters, and 9 had words. These will undoubtedly be the types of synesthesia interfering most strongly with the sound of a voice. The influence of words on synesthetic perceptions could have been avoided by using nonsense syllables. However, some word synesthetes also experience concurrents with nonsense words. Moreover, if the interference of words as an inducer had been overwhelmingly strong, participants would have reported the same associations for all stimuli containing the same sentence. This scenario was not found. The complexity of voice as an inducer and its tendency to coexist with other types are, in our opinion, the most likely cause of the low consistency scores found in this experiment, and underlines the necessity both for further research on this type of synesthesia, and for redefinition of the role of consistency in the definition of synesthesia, as discussed in the next section.

### Theoretical context and implications

Results of the verbal descriptions showed a clear distinction of the use of synesthetic terms between synesthetes and non-synesthetes: the former use more synesthetic terms than the latter. It cannot be excluded that this result may be biased since self-reported synesthetes may be more explicit in naming synesthetic associations because of their knowledge that they were selected for this experiment *as* voice-induced synesthetes.

In light of the findings of weak consistency within synesthetes and shared results across participant groups, the question arises whether voice-induced synesthesia is in fact a clearly defined and distinct variant of synesthesia at all. Furthermore, it is also possible that voice-color synesthesia may be nothing more than an epiphenomenon of *music*-color synesthesia. Indeed, many of our synesthetes had both types of synesthesia co-existing together suggesting they may be one and the same phenomenon. However, two facts argue against this: first, the co-existence of both forms is not in itself a reason to dismiss voice-color synesthesia, and this is because different variants of synesthesia do tend to co-exist together within the same individual (i.e., even if they are separate forms; see e.g., Simner et al., 2006; Novich et al., 2011). More importantly, we found at least one case (LP) of a synesthete reporting voice-color synesthesia *without* the music-color variant. It is possible therefore that these forms do exist as separate conditions.

Traditionally, low consistency rates define somebody as a non-synesthete (Asher et al., 2006; Ward and Mattingley, 2006). With new approaches, however, this rigid definition faces revision. There is a risk of circularity in defining synesthesia by its consistency over time: If non-consistent synesthetes are not defined as synesthetes, consistency becomes a defining criterion (Cohen Kadosh and Terhune, 2012; Eagleman, 2012; Simner, 2012a,b). Recent studies have shown that consistency cannot be used as a proof of genuineness for all variants of synesthesia, nor in fact for all synesthetes (Simner et al., 2011; Simner and Ludwig, 2012). With the suggestion of introducing a synesthesia spectrum (Eagleman, 2012) it becomes apparent that one can be more or less synesthetic. Based on this suggestion there are two speculations on how to define our synesthete participants. First—considering consistency as a defining criterion—they could be “moderately synesthetic,” Second, we could further speculate that a spectrum exists not only within types of synesthesia but also in terms of the influence of neighboring types. Music-induced synesthetes for example may show an increased likelihood of additionally having voice-induced synesthesia to a certain degree, but may be more or less consistent in their associations of this additional type.

It can be concluded that most results of our study show similarities across participant groups, feeding into the discussion that being synesthetic lies on a continuum (Eagleman, 2012; Simner, 2012b). This suggests common underlying mechanisms in associations, which synesthetes access at a conscious and non-synesthetes at a subconscious level. The indications in our results of individual differences in descriptive use of synesthetic terms as well as in consistency suggest that more emphasis should be put on these differences within synesthetes, and they should be taken into account when “classifying” synesthetes. In fact, a categorization of synesthetes and non-synesthetes might not be achievable in the same way for voice synesthesia as for other variants of the condition; the entanglement of multiple types of synesthesia within one individual must be taken into account in future research seeking to develop a fuller understanding of the role of voice as an inducer.

### Conflict of interest statement

The authors declare that the research was conducted in the absence of any commercial or financial relationships that could be construed as a potential conflict of interest.
